# Chinese Medicine as an Adjunctive Treatment for Gastric Cancer: Methodological Investigation of meta-Analyses and Evidence Map

**DOI:** 10.3389/fphar.2021.797753

**Published:** 2022-01-10

**Authors:** Cuncun Lu, Lixin Ke, Jieyun Li, Shuilin Wu, Lufang Feng, Youyou Wang, Alexios Fotios A. Mentis, Peng Xu, Xiaoxiao Zhao, Kehu Yang

**Affiliations:** ^1^ Evidence-Based Medicine Center, School of Basic Medical Sciences, Lanzhou University, Lanzhou, China; ^2^ Evidence-Based Social Science Center, School of Public Health, Lanzhou University, Lanzhou, China; ^3^ Key Laboratory of Evidence-Based Medicine and Knowledge Translation of Gansu Province, Lanzhou, China; ^4^ Institute of Basic Research in Clinical Medicine, China Academy of Chinese Medical Sciences, Beijing, China; ^5^ Hepatobiliary and Pancreatic Center, The First Affiliated Hospital, Sun Yat-sen University, Guangzhou, China; ^6^ National Resource Center for Chinese Materia Medica, China Academy of Chinese Medical Sciences, Beijing, China; ^7^ University Research Institute of Maternal and Child Health and Precision Medicine, National and Kapodistrian University of Athens, Athens, Greece

**Keywords:** traditional Chinese medicine, herbal medicine, gastric cancer, meta-analyses, methodological quality, AMSTAR-2, efficacy, safety

## Abstract

**Background:** Many meta-analyses (MAs) on Chinese medicine (CM) as an adjunctive treatment for gastric cancer have been published in recent years. However, the pooled evidence reported in MAs and their methodological quality remain unknown. Therefore, we designed a study to comprehensively evaluate and summarize the current evidence of CMs for gastric cancer in published MAs.

**Methods:** A systematic search on MAs published in English from inception to 1st September 2021 was conducted in PubMed and Embase. The AMSTAR-2 tool was used to evaluate the methodological quality of the included MAs, and the results of the quality assessment were visualized using the evidence mapping method. Stata 17/SE was used for statistical analysis (Registration number: INPLASY202190005).

**Results:** A total of 20 MAs (16 pairwise and 4 network MAs) were included from 118 records. These MAs were published in 14 journals from 2013 to 2021, with the number of patients and trials ranging from 688 to 6,857, and from 10 to 85, respectively. A large number of CMs (e.g., AiDi, FuFangKuShen, and HuaChanSu) in combination with chemotherapy for gastric cancer were identified among the included MAs. According to the pooled results reported in MAs, when compared to chemotherapy alone, CMs in combination with chemotherapy not only improve various outcomes on efficacy (e.g., objective response rate, quality of life) but also reduce various adverse reactions (e.g., leucopenia, nausea and vomiting). Only 2 MAs were low in terms of the overall methodological quality, while the other 18 MAs were all critically low. The methodology was required to be advanced significantly, mainly involving: study protocol and registration, explanation for the inclusion of study design, list of excluded studies with justifications, adequate details of included studies, reporting on funding sources of primary studies, and evaluation of the potential impact of risk of bias. In addition, MAs that received funds support (β = 2.68; 95%CI: 0.40 to 4.96; p = 0.024) or were published in journals with higher impact factor (β = 2.81; 95%CI: 0.69 to 4.92; p = 0.012) had a higher score on the overall methodological quality in the univariate analysis, but the results were not statistically significant according to the multivariate analysis.

**Conclusion:** Combining CMs with chemotherapy can potentially improve clinical outcomes and reduce the relevant adverse effects in patients with gastric cancer. However, the methodological quality of relevant MAs requires significant improvement, and the current evidence needs to be validated through multinational trials that are well-designed and have a large sample size.

## Introduction

Gastric cancer and other cancers seriously affect patients’ health and quality of life (Sung et al., 2021). According to the latest cancer statistics report (Sung et al., 2021), gastric cancer is the fifth most commonly diagnosed cancer, with an estimated 1.09 million new cases (5.63%) on a global scale, and it is the fourth leading cause of death related to cancer, with an estimated 0.77 million death cases (7.72%). Although there are many therapeutic modalities (e.g., surgery, chemotherapy, and immunotherapy) available for gastric cancer, unfortunately, most cases are already at an advanced stage at the time of diagnosis and/or detection (Cheng et al., 2021). Currently, chemotherapy remains one of the most important therapies for gastric cancer (Li et al., 2020). However, patients with gastric cancer have developed resistance to chemotherapy, as noted in daily clinical practice (Li et al., 2020; Cheng et al., 2021). Furthermore, chemotherapy is known to cause severe adverse reactions or side effects (Li et al., 2020; Cheng et al., 2021).

Chinese medicine (CM) is a personalized therapy for the treatment of human cancers (Wang et al., 2018) and has been widely used to treat gastric cancer in China and other Asian countries (Li et al., 2015). In China, clinicians typically employ CMs in combination with chemotherapy to improve the efficacy of treatment in patients with gastric cancer, while decreasing the adverse drug reactions caused by chemotherapy (Cheng et al., 2021). Nevertheless, the very notion of evidence-based medicine (EBM) emphasizes that all clinical decisions should be made based on the best available evidence (Lu et al., 2019). A systematic review with meta-analysis (MA) is widely recognized as the highest level of evidence in the EBM field (Abushouk et al., 2021), but the reliability of pooled results reported in MAs is often hampered by methodological weaknesses (Ioannidis, 2016). Furthermore, redundant and conflicting MAs on the same topic can confuse clinicians (Chapelle et al., 2021), even leading to clinical decision-making errors and secondary harms to the relevant patients.

In recent years, a large number of MAs (Chen et al., 2018; Chen et al., 2020; Li et al., 2020; Wu et al., 2020; Cheng et al., 2021) focusing on CM as an adjunctive treatment for gastric cancer have been published. For example, Cheng *et al.* (Cheng et al., 2021) published a systematic review with MA, focusing on the efficacy and safety of CMs containing astragalus combined with platinum-based chemotherapy for advanced gastric cancer. In 2020, Li *et al.* (Li et al., 2020) conducted an MA on paclitaxel-based chemotherapy in combination with CMs for gastric cancer. Chen *et al.* (Chen et al., 2020) used an MA to summarize the efficacy of the SiJunZi decoction combined with enteral nutrition for the treatment of gastric cancer. Although many relevant systematic reviews with MAs have been published, no research has synthesized the evidence reported in published MAs that focused on the efficacy and safety of CMs combined with other treatments (e.g., chemotherapy and nutritional intervention) for gastric cancer, as well as evaluated the methodological quality of these MAs.

Therefore, based on our previous study (Lu et al., 2021), we designed a methodological overview to fill the aforementioned pending knowledge gaps. Specifically, this methodological research had two main objectives: 1) using the A Measurement Tool to Assess Systematic Reviews 2 (AMSTAR-2) (Shea et al., 2017) tool to identify methodological weaknesses in published MAs that focused on CM as an adjunctive treatment for gastric cancer, doing so can contribute to improving the design, implementation and reporting of future relevant MAs; 2) comprehensively summarizing available evidence on the efficacy and safety of CMs combined with other treatments of gastric cancer, providing evidence support to aid clinician decision-making, and developing clinical practice guidelines, especially in settings where CMs are heavily practiced.

## Methods

### Study Registration and Reporting

This methodological overview of MAs for CMs for gastric cancer has been registered on the International Platform of Registered Systematic Review and Meta-analysis Protocols website (http://inplasy.com/; registration number: INPLASY202190005). The current study was reported in accordance with the Preferred Reporting Items for Systematic reviews and Meta-Analyses (PRISMA) checklist (Liberati et al., 2009) (Supplementary Material S1). Ethical approval and patient consent were waived since this study was an overview based on published documents.

### Literature Search

PubMed and Embase, two commonly used databases, were systematically searched for published MAs focusing on CMs for patients with gastric cancer. The search timeframe was set from inception to 1st September 2021. In order to identify all potentially relevant publications available in English, Medical Subject Headings terms, and free-text words were used, such as “Medicine, Chinese Traditional,” “Complementary Therapies,” “Chinese medicine,” “Traditional medicines,” “Decoction,” “San,” “Herb*,” “Pill*,” “Formula*,” “Granule*,” “Injection*,” “Stomach Neoplasms,” “Gastric cancer,” “Gastric carcinoma,” “Gastric neoplasm,” “Stomach cancer,” “Stomach tumor,” “Stomach adenocarcinoma,” “Systematic review,” and “Meta-analysis,” *etc.* The reference lists of the included MAs were also checked to identify potential MAs that may have been missed by the database search. The details of the search strategy are shown in Supplementary Material S2.

### Eligibility Criteria

Studies that met the following criteria were included: 1) type of studies: peer-reviewed pairwise or network MAs (without restriction on the study design of primary studies) published in English, and with the definition of MAs used in this methodological overview consistent with our previous publication (Lu et al., 2021); 2) type of participants: patients with gastric cancer confirmed by histology and/or imaging examination, regardless of age, sex, tumor stage, educational background, socioeconomic status, nationality, and race/ethnicity; 3) type of interventions: ① CM combined with chemotherapy *vs* chemotherapy or other CMs alone, ② CM *vs* chemotherapy, ③ comparison of different CMs, without formulation, dosage, usage, and treatment duration restrictions; 4) type of clinical outcomes: any pooled clinical outcome related to efficacy or safety reported in the included MAs was considered appropriate.

Conference abstracts, comments, protocols, duplications, primary studies, qualitative reviews (e.g., traditional expert review, qualitative systematic review, and overview), methodological papers, the old version of the Cochrane review, MAs focusing on the treatment of postoperative complications (e.g., dumping syndrome) or containing other traditional remedial regimens (e.g., acupuncture, moxibustion, or tai chi), retracted articles, and non-English publications were excluded.

### Study Selection and Data Extraction

For the selection of eligible studies, all identified hits were exported from PubMed and Embase and imported into Endnote (Version X9, Clarivate Analytics). First, duplicate records were removed manually and by software. Next, the titles and abstracts of the remaining records were examined to exclude irrelevant documents. Finally, the full texts of the rest of the studies were retrieved for further screening. After completing the study selection, the following information was abstracted from the included MAs: title, first author, publication year, journal with impact factor (IF_2020_), country of the corresponding author, “mention of PRISMA” (meaning an MA claimed that it was carried out according to the PRISMA or other reporting guidelines; the default answer is “Yes” for a Cochrane review as its reporting is detailed), protocol and registration, search sources, patients’ information, intervention and comparison, number and types of included original studies, the total number of patients, quality assessment tool of included primary studies, information on funds support, use of GRADE (Grading of Recommendations Assessment, Development and Evaluation) (Schwingshackl et al., 2021), pooled effect sizes (e.g., RR [Risk Ratio], OR [Odds Ratio], and MD [Mean Difference]) with heterogeneity indexes, and key results.

Study selection and data extraction were performed by two independent investigators (L. Ke and J. Li). Any discrepancy was resolved through discussion or consultation with the leading author (C. Lu or K. Yang).

### Assessment of Methodological Quality

The AMSTAR-2 (Shea et al., 2017) is a widely recognized tool (Lu C. et al., 2020; Pieper et al., 2021) for assessing the methodological quality of systematic reviews and MAs of healthcare interventions. This tool consists of 16 items (Shea et al., 2017), seven of which (items 2, 4, 7, 9, 11, 13, and 15) being critical items. For the leading question of these items, the “Yes” (Y), “Partial Yes” (PY), or “No” (N) options were used to assess the compliance of included MAs; in order to facilitate statistical analysis, “Y,” “PY,” and “N” were correspondingly scored 1, 0.5, and 0 points for the non-critical items, and 2, 1, and 0 points for the critical items. Finally, the overall confidence of each MA was classified as “Critically Low” (CL, “*more than one critical flaw with or without non-critical weaknesses*”), “Low” (L, “*one critical flaw with or without non-critical weaknesses*”), “Moderate” (M, “*more than one non-critical weakness*”), or “High” (H, “*No or one non-critical weakness*”) (Shea et al., 2017). Two reviewers (C. Lu and L. Ke) with a background in EBM, employed this tool to independently evaluate the methodological quality of the included MAs, while any further disagreement was resolved through detailed discussions.

### Statistical Analysis

The basic characteristics of the included MAs and pooled clinical outcomes of interest were qualitatively described. The adherence of the AMSTAR-2 tool was presented as a number and percentage with a 95% confidence interval (95% CI) according to three options: “Y,” “PY,” and “N.” The univariate and multivariate linear regression analyses were used to examine whether any study characteristics (i.e., PRISMA mention, journal’s IF, funds support) potentially influenced the overall methodological quality score. The multicollinearity was not obvious when variance inflation factor (VIF) was less than 6 (Li et al., 2019). The evidence mapping method was used to visualize the overall methodological quality for each of MA (Chen et al., 2021; Lu et al., 2021). In an evidence map, each bubble represented a publication, with the size of the bubble proportional to the total number of patients included in each MA, the color showing the types of MA (i.e., pairwise or network MA), the *X*-axis indicating the overall confidence (i.e., “H,” “M,” “L,” “CL”) according to the AMSTAR-2 tool, and the *Y*-axis indicating the publication year of each MA. Spider charts and forest plots were also used to display the results. Data analysis was performed using Stata 17/SE (StataCorp, College Station, TX, United States) and Excel 2016 (Microsoft Corporation, WA, United States). A two-sided *p* < 0.05 was considered statistically significant.

## Results

### Search and Selection

**FIGURE 1:**
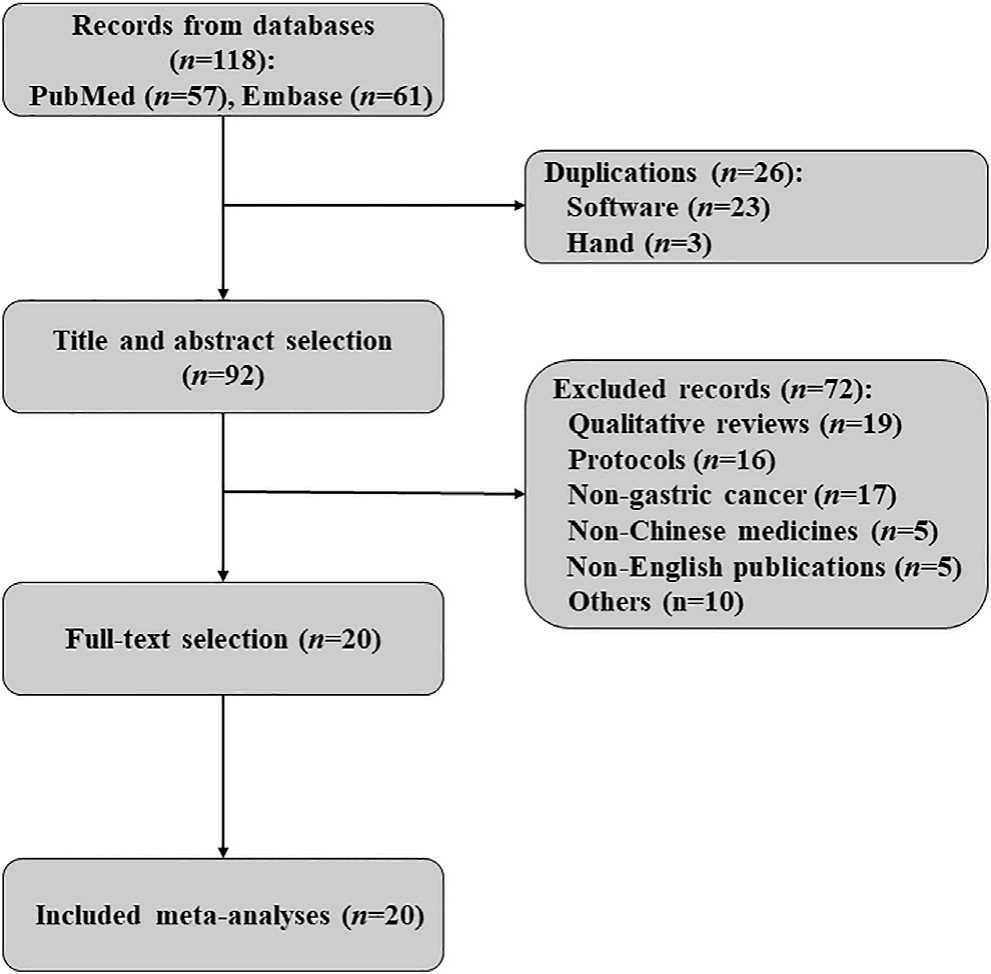
Flow chart of MAs selection for the methodological overview.

A total of 118 records were obtained from PubMed (*n* = 57) and Embase (*n* = 61). After removing 26 duplicates, 92 titles and abstracts were further screened. Finally, 20 systematic reviews with MAs (Xie et al., 2013; Yang et al., 2013; Li et al., 2014; Wang et al., 2014; Yao et al., 2014; Li et al., 2015; Wang et al., 2015; Zhang et al., 2017; Zhang D. et al., 2018; Zhang X. et al., 2018; Chen et al., 2018; Lee et al., 2018; Wu et al., 2018; Sun et al., 2019; Wu et al., 2019; Lu X. et al., 2020; Chen et al., 2020; Li et al., 2020; Wu et al., 2020; Cheng et al., 2021) in English that focused on CM for gastric cancer were included (no different MA was identified from the reference lists). The selection flow used in this research is displayed in Figure 1.

### Basic Characteristics of Included MAs

The 20 systematic reviews (16 pairwise MAs and 4 network MAs) included in this overview mainly involved various CMs (e.g., AiDi, FFKS, HuaChanSu, KangAi, and SQFZ were all reported by five or more MAs) combined with chemotherapy or enteral nutrition to treat gastric cancer. These MAs were published by two countries, including China (*n* = 19, 95.00%) and Korea (*n* = 1, 5.00%); the years of publication ranged from 2013 to 2021, with most studies (*n* = 5, 25.00%) published in 2018. In terms of journals, 20 MAs were published in 14 peer-reviewed journals, with *Evidence-Based Complementary and Alternative Medicine* (IF_2020_ = 2.629) publishing 5 MAs (25.00%) and was ranked first, while *Cochrane Database of Systematic Reviews* (IF_2020_ = 9.266), *Frontiers in Oncology* (IF_2020_ = 6.244), *Frontiers in Pharmacology* (IF_2020_ = 5.81), and *OncoTargets and Therapy* (IF_2020_ = 4.147) ranked highly in terms of IFs. The most common software tools used for conducting MAs were RevMan (*n* = 14, 70.00%) and Stata (*n* = 11, 55.00%).

There are eight databases (PubMed/Medline, Embase, Cochrane Library, Web of Science, CNKI, CBM, WanFang, and VIP) that are most commonly used to conduct a literature search for published systematic reviews of traditional CMs. The MAs included in our overview searched 4 to 8 databases, with the most common English databases being PubMed/Medline and Cochrane Library (*n* = 20, 100%), and the most common Chinese databases being CNKI (*n* = 19, 95.00%) and WanFang (*n* = 16, 80.00%). All included MAs reported that only randomized controlled trials (RCTs) or quasi trials were included, and the most commonly used tool for assessing the quality or risk of bias was the previous version of the Cochrane tool (*n* = 17, 85.00%) (Schwingshackl et al., 2021). The total sample size of each MA ranged from 688 to 6,857 patients, with an average of around 2,062 patients, and the number of trials included ranged from 10 to 85. Ten (50.00%) MAs explicitly claimed that they followed the PRISMA or Quality of Reporting of Meta-analyses guidelines (QUOROM) (Pussegoda et al., 2017) while conducting or reporting their studies, and 13 (65.00%) MAs acknowledged that they received the funds support. Only two (10.00%) systematic reviews registered their protocols and employed the GRADE to evaluate the evidence quality based on clinical outcomes. The details of the basic characteristics of included MAs are shown in Table 1.

**TABLE 1:** Basic characteristics of the included MAs.

Study	Country	Journal and IF_2020_	Patient	Intervention	Types of MA	Software	PRISMA mention	Protocol and registration	Databases	Sample size	Trials	Tool for quality assessment	Funds	GRADE
Xie et al. (2013)	China	Medical Hypotheses, 1.538	Advanced gastric cancer (III-IV)	HuaChanSu + chemotherapy vs Chemotherapy	Pairwise	Stata	QUOROM	NM	PubMed/Medline, Cochrane Library, CNKI, CBM, WanFang, VIP	1,008	15	Self-developed tool	Yes	NM
Yang et al. (2013)	China	Cochrane Database of Systematic Reviews, 9.266	Advanced gastric cancer (III-IV)	CM (AiDi, HuaChanSu, FFKS, SQFZ, *etc.*)+western therapy (e.g., chemotherapy) vs Western therapy, CM (BanXia, RenShen, etc.)+Western therapy vs CM, CM1 (HuaChanSu, GanQi, etc.) vs CM2, CM (DangShen, SQFZ, *etc.*) vs Western therapy	Pairwise	RevMan	Yes	Cochrane Library	PubMed/Medline, Embase, Cochrane Library, CBM	6,857	85	Cochrane tool 1.0	Yes	Yes
Li et al. (2014)	China	Journal of Cancer Research and Therapeutics, 1.805	Gastric cancer	FFKS injection 20 ml/d + chemotherapy vs Chemotherapy	Pairwise	Stata	No	NM	PubMed/Medline, Embase, Cochrane Library, CNKI	1,061	13	No	No	NM
Wang et al. (2014)	China	Asian Pacific Journal of Cancer Prevention, _	Advanced gastric cancer	10 CM injections (AiDi 50–100ml, Astragalus polysaccharide 250ml, HuaChanSu 10–20ml, FFKS 15–20ml, DeLiSheng 40ml, Ginseng polysaccharide 24ml, KangAi 30–60ml, KangLaiTe 100–200ml, SQFZ 250ml, BJOE 30 ml)+FOLFOX vs FOLFOX	Network	ADDIS	No	NM	PubMed/Medline, Embase, Cochrane Library, Web of Science, CNKI, CBM, WanFang, VIP	2,761	38	Cochrane tool 1.0, CONSORT 2010	NM	NM
Yao et al. (2014)	China	Journal of Cancer Research and Therapeutics, 1.805	Advanced gastric cancer	SQFZ injection + chemotherapy vs Chemotherapy	Pairwise	MetaAnalyst	No	NM	PubMed/Medline, Embase, Cochrane Library, CNKI	1,621	15	Jadad	No	NM
Wang et al. (2015)	China	Journal of Traditional Chinese Medicine, 0.848	Gastric cancer	AiDi injection 50–100 ml + chemotherapy vs Chemotherapy	Pairwise	RevMan	No	NM	PubMed/Medline, Embase, Cochrane Library, Web of Science, CNKI, CBM, WanFang, VIP	1927	32	Cochrane tool 1.0, CONSORT 2010	NM	NM
Li et al. (2015)	China	Chinese Journal of Integrative Medicine, 1.978	Advanced gastric cancer	SQFZ injection 250 ml/d + chemotherapy vs Chemotherapy	Pairwise	RevMan	No	NM	PubMed/Medline, Embase, Cochrane Library, Web of Science, CNKI, CBM, WanFang	860	13	Cochrane tool 1.0, Jadad	NM	NM
Zhang et al. (2017)	China	Oncotarget, _	Gastric cancer	15 CM injections (AiDi 50–100 ml, Astragalus polysaccharide 250 mg, Astragalus 50 ml, HuaChanSu 10–50 ml, Disodium cantharidinate and vitamin B6 40 ml, DeLiSheng 40 ml, Elemene 100 ml, FFKS 15–30 ml, Ginseng polysaccharide 12–24 mg, KangAi 30–60 ml, Lentinan 1–12 mg, Placental polypeptide 8 ml, ShenMai 40 ml, SQFZ 250 ml, XiaoAiPing 80ml/60 mg)+FOLFOX vs FOLFOX	Network	Stata, Winbugs	Yes	NM	PubMed/Medline, Embase, Cochrane Library, CNKI, CBM, WanFang, VIP	5,978	81	Cochrane tool 1.0, CONSORT 2010	Yes	NM
Chen et al. (2018)	China	Evidence-Based Complementary and Alternative Medicine, 2.629	Gastric cancer (I-IV)	JPBS therapy (BSJP decoction, BSJP oral liquid, FFEJ Jiang, JPBS decoction)+chemotherapy vs Chemotherapy	Pairwise	RevMan	No	NM	PubMed/Medline, Embase, Cochrane Library, CNKI, WanFang, VIP	3,098	26	Cochrane tool 1.0	Yes	NM
Lee et al. (2018)	Korea	Integrative Cancer Therapies, 3.279	Resectable gastric cancer	CM (Oral decoction/pill/capsule: BaiHuaSheSheCao + BanZhiLian, BieJiaJian Wan, BaiHuaSheSheCao + YiYiRen, Fulin + YiYiRen, FuLin + BaiZhu, FuLin + BaiZhu + TaiZiShen, Norcantharidin)+Postsurgical chemotherapy vs Postsurgical chemotherapy	Pairwise	RevMan	No	NM	PubMed/Medline, Embase, Cochrane Library, CNKI	1,075	13	Cochrane tool 1.0	Yes	NM
Wu et al. (2018)	China	Evidence-Based Complementary and Alternative Medicine, 2.629	Gastric cancer	BJOE injection 20–30 ml/d + chemotherapy vs Chemotherapy	Pairwise	RevMan, Stata	No	NM	PubMed/Medline, Embase, Cochrane Library, CNKI, CBM, WanFang, VIP	912	13	Cochrane tool 1.0	Yes	NM
Zhang D. et al. (2018)	China	Medicine, 1.889	Gastric cancer	13 CM injections (Aidi 50–100 ml, Astragalus polysaccharide 50 mg, BJOE 20–30 ml, HuaChanSu 30 ml, Disodium cantharidinate and vitamin B6 30 ml, Elemene 100 mg, Lentinan 1 mg, FFKS 20 ml, SQFZ 250 ml, KangAi 40–80 ml, ShenFu 50 ml, ShenMai 60 ml, XiaoAiPing 40–60 ml)+XELOX vs XELOX	Network	Stata, Winbugs	Yes	NM	PubMed/Medline, Embase, Cochrane Library, CNKI, CBM, WanFang, VIP	2,154	26	Cochrane tool 1.0	Yes	NM
Zhang X. et al. (2018)	China	Evidence-Based Complementary and Alternative Medicine, 2.629	Advanced gastric cancer (III-IV)	HuaChanSu injection 10–50 ml + chemotherapy vs Chemotherapy	Pairwise	RevMan, Stata	Yes	NM	PubMed/Medline, Embase, Cochrane Library, CNKI, WanFang, VIP	853	12	Cochrane tool 1.0	Yes	NM
Sun et al. (2019)	China	OncoTargets and Therapy, 4.147	Advanced gastric cancer	HuaChanSu 10–50ml/200–1200 mg + chemotherapy vs Chemotherapy	Pairwise	RevMan, Stata	Yes	NM	PubMed/Medline, Embase, Cochrane Library, Web of Science, CNKI, CBM, WanFang, VIP	1939	27	Cochrane tool 1.0	No	NM
Wu et al. (2019)	China	Evidence-Based Complementary and Alternative Medicine, 2.629	Advanced gastric cancer	XiaoAiPing injection 40–80 ml/60 mg + chemotherapy vs Chemotherapy	Pairwise	RevMan, Stata	No	NM	PubMed/Medline, Embase, Cochrane Library, CNKI, CBM, WanFang, VIP	1,097	14	Cochrane tool 1.0	Yes	NM
Chen et al. (2020)	China	Nutrition and Cancer, 2.9	Gastric cancer	SiJunZi decoction + enteral nutrition vs Enteral nutrition	Pairwise	RevMan	Yes	NM	PubMed/Medline, Embase, Cochrane Library, Web of Science, CNKI, CBM, WanFang, VIP	688	10	Cochrane tool 1.0	No	NM
Li et al. (2020)	China	Frontiers in Pharmacology, 5.81	Advanced gastric cancer (III-IV)	CM (Injection: AiDi, HuaChanSu, FFKS, FFKS + YQYW decoction, KangAi, KangLaiTe + JPYQ decoction; Oral: FFBM capsule, FZHW liquid medicament, JPXZ decoction, LiuJunZi decoction, Rg3, Rg3+ShenYi capsule, SLBZ decoction)+Paclitaxel-based chemotherapy vs Paclitaxel-based chemotherapy	Pairwise	RevMan	No	NM	PubMed/Medline, Embase, Cochrane Library, CNKI, WanFang, VIP	1,109	14	Cochrane tool 1.0, Jadad	Yes	NM
Lu X. et al. (2020)	China	Evidence-Based Complementary and Alternative Medicine, 2.629	Gastric cancer	CM (Oral Chinese patent medicine: AnTiKe capsule 0.44 g, BaZhen granule 3.5 g, HuaChanSu capsule 0.5–0.9 g, PingXiao capsule 1.15–1.84 g, SQSYW granule 2 g, XiaoAiPing tablet 1.8–3 g, ZQFZ granule 5–15 g)+chemotherapy vs Chemotherapy	Network	RevMan, Stata, ADDIS	Yes	NM	PubMed/Medline, Embase, Cochrane Library, CNKI, CBM, WanFang, VIP	2,602	30	Cochrane tool 1.0	Yes	NM
Wu et al. (2020)	China	Journal of Traditional Chinese Medicine, 0.848	Gastric cancer	HuaChanSu injection 10–50 ml + chemotherapy vs chemotherapy	Pairwise	RevMan, Stata	Yes	NM	PubMed/Medline, Embase, Cochrane Library, CNKI, CBM, WanFang, VIP	976	14	Cochrane tool 1.0	Yes	NM
Cheng et al. (2021)	China	Frontiers in Oncology, 6.244	Advanced gastric cancer (III-IV)	CM containing Astragalus (Injection: AiDi 40–80 ml/d, DeLiSheng 40 ml/d, KangAi 60 ml/d, SQFZ 250 ml/d; Oral: Astragalus-based formula 200–400 ml/d, CiDan capsule 5.4 g/d, BoErNing capsule 1.8 g/d, WeiNing granule 400 ml/d)+Platinum-based chemotherapy vs Platinum-based chemotherapy	Pairwise	RevMan, Stata	Yes	PROSPERO, CRD42020203486	PubMed/Medline, Embase, Cochrane Library, CNKI, CBM, WanFang, VIP	2,670	35	Cochrane tool 1.0	Yes	Yes

Note: BJOE, brucea javanica oil emulsion; BSJP, BuShenJianPi; CM, chinese medicine; CBM, chinese biomedical literature Database; CNKI, china national knowledge infrastructure Database; CONSORT, consolidated standards of reporting trials; FFBM, FuFangBanMao; FFEJ, FuFangEJiao; FFKS, FuFangKuShen; FOLFOX, 5-Fluorouracil combined with Leucovorin and Oxaliplatin; FZHW, FuZhengHeWei; GRADE, grading of recommendations assessment, Development and Evaluation; IF, impact factor; JPBS, JianPiBushen; JPYQ, JianPiYiQi; JPXZ, JianPiXiaoZheng; MAs, Meta-analyses; NM, not mentioned; PRISMA, Preferred Reporting Items for Systematic reviews and Meta-Analyses guidelines; PROSPERO, international prospective register of systematic reviews; QUOROM, Quality of Reporting of Meta-analyses guidelines; SLBZ, ShenLingBaiZhu; SQFZ, ShenQiFuZheng; SQSYW, ShenQiShiYiWei; XELOX, capecitabine combined with oxaliplatin; VIP, china science and technology journal Database; YQYW, YiQiYangWei. The abbreviations of some nouns used in the results, discussions, and conclusions were consistent with here, therefore the full names of these nouns were omitted in these sections.

### Methodological Quality of Included MAs

**FIGURE 2:**
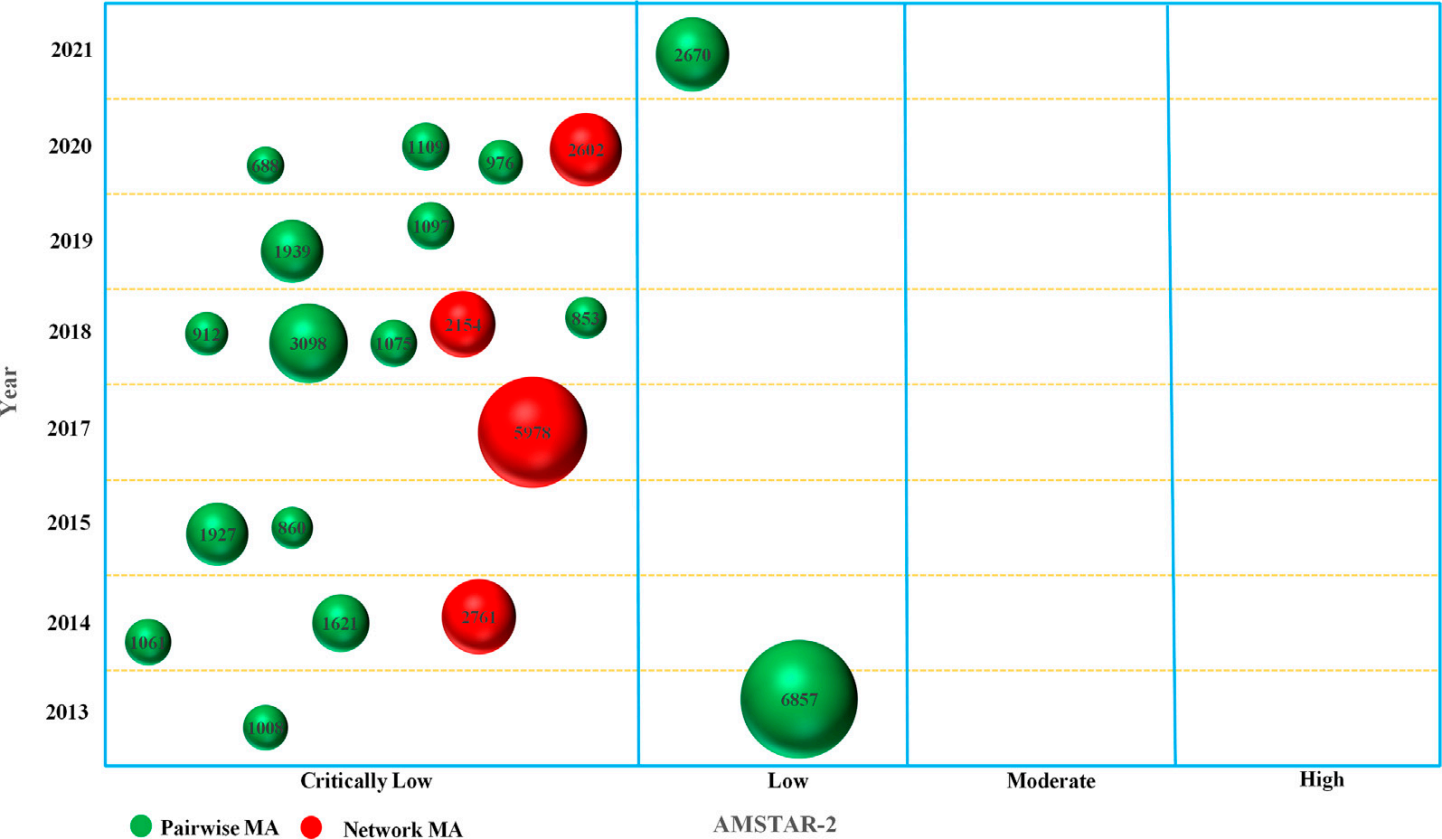
Bubble plot of the overall methodological quality.

**FIGURE 3:**
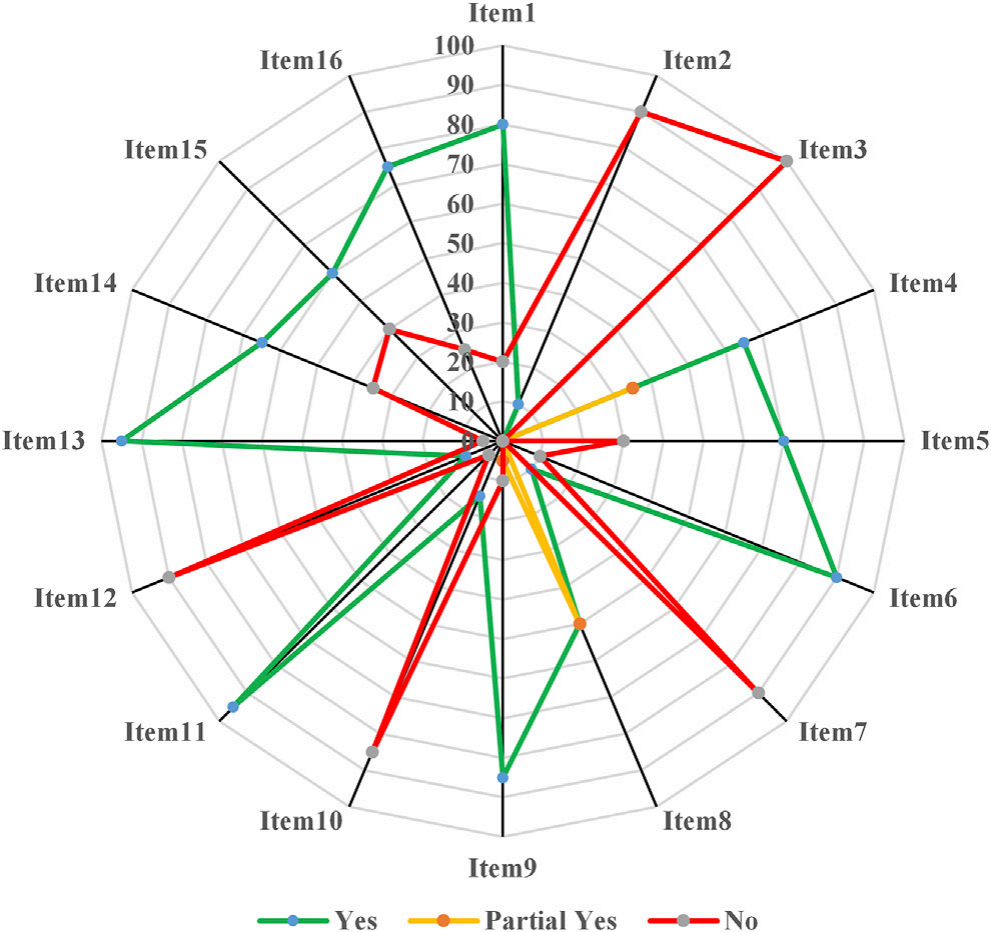
Methodological quality based on the AMSTAR-2 tool.

Based on the AMSTAR-2, only 2 MAs were rated as “L” in terms of the overall methodological quality, while the remaining 18 MAs were all graded as “CL” (Figure 2, Supplementary Material S3). Specifically (Figure 3), for “*item 1. Did the research questions and inclusion criteria for the review include the components of participant, intervention, comparison, and outcome,*” 16 (80.00%, 95% CI: 38.66%–78.12%) MAs were assessed as “Y,” while 20.00% (4/20, 95% CI: 22.00%–26.25%) were “N.” Only 2 MAs (10.00%, 95% CI: 2.79%–30.10%) that provided information on study protocol and registration were evaluated as “Y” in “item 2,” whereas the others (90.00%, 95% CI: 69.90%–97.21%) were all rated as “N.” As none of the MAs explained the reason for the inclusion of the study design, all of them were evaluated as “N” in “item 3.” For “item 4. Did the review authors use a comprehensive literature search strategy,” 65.00% (13/20, 95% CI: 43.29%–81.88%) of the MAs were evaluated as “Y” because they conducted supplemental retrieval, such as reference lists, while the remaining seven (35.00%, 95% CI: 18.12%–56.71%) MAs were assessed as “P.”

The study selection was performed in duplicate by 70.00% (14/20, 95% CI: 48.10%, 85.45%) MAs, which were assessed as “Y” in “item 5,” while six (30.00%, 95% CI: 14.55%–51.90%) MAs were evaluated as “N.” For “*item 6. Did the review authors perform data extraction in duplicate,*” 18 (90.00%, 95% CI: 69.90%–97.21%) MAs were “Y,” while 10.00% (2/20, 2.79%–30.10%) were assessed as “N.” Two (10.00%, 95% CI: 2.79%–30.10%) MAs were rated as “Y” because they provided a list of excluded studies and corresponding reasons, while 18 MAs (90.00%, 95% CI: 69.90%–97.21%) were evaluated as “N” in “item 7.” Half of included MAs were identified as “N” because they did not provide any information on age, sex, or dosage of CMs, among others, while the other half was evaluated as “P” in “item 8.” The risk of bias in trials was evaluated by 85.00% (17/20, 95% CI: 63.96%–94.76%) using the Cochrane tool and the MAs were thus assessed as “Y” in “item 9”; one (5.00%, 95% CI: 0.89%–23.61%) MA was evaluated as “P” as it only it used Jadad scale, while the others (2/20, 95% CI: 2.79%–30.10%) were appraised as “N.” Only three (15.00%, 95% CI: 5.24%–36.04%) MAs were classified as “Y” in “item 10,” as they reported the funding information of RCTs in the results, whereas the others (17/20, 95% CI: 22.00%–26.25%) were assessed as “N.”

For “*item 11. If meta-analysis was performed did the review authors use appropriate methods for statistical combination of results,*” 19 (95.00%, 95% CI: 76.39%–99.11%) MAs were assessed as “Y,” the remaining one (5.00%, 95% CI: 0.89%–23.61%) was evaluated as “N.” Only two (10.00%, 95% CI: 2.79%–30.10%) MAs assessed the potential impact of risk of bias in individual trials on the pooled results, then evaluated as “Y” in “item 12,” while 90.00% (18/20, 95% CI: 69.90%–97.21%) of MAs were assessed as “N.” For “*item 13. Did the review authors account for risk of bias in individual studies when interpreting/discussing the results of the review,*” 19 (95.00%, 95% CI: 76.39%–99.11%) MAs were appraised as “Y,” while one (5.00%, 95% CI: 0.89%–23.61%) was evaluated as “N.” Thirteen (65.00%, 95% CI: 43.29%–81.88%) MAs satisfactorily explained and discussed the heterogeneity and were assessed as “Y” in “item 14,” but the remaining seven (35.00%, 95% CI: 18.12%–56.71%) MAs were evaluated as “N.” Of note, 40.00% (8/20, 95% CI: 21.88%, 61.34%) MAs were assessed as “N” since they did not investigate publication bias or discuss its potential impact adequately, and 60.00% (12/20, 95% CI: 18.12%–56.71%) were rated as “Y” in “item 15.” Fifteen MAs (75.00%, 95% CI: 53.13%–88.81%) stated that they had no conflicts of interest and were evaluated as “Y” in “item 16,” while the others (25.00%, 95% CI: 11.19%–46.87%) were appraised as “N.” Overall, the methodology of the included MAs had considerable flaws (percentage of “Y” < 60%) in items 2, 3, 7, 8, 10, and 12 of the AMSTAR-2 tool.

**FIGURE 4:**
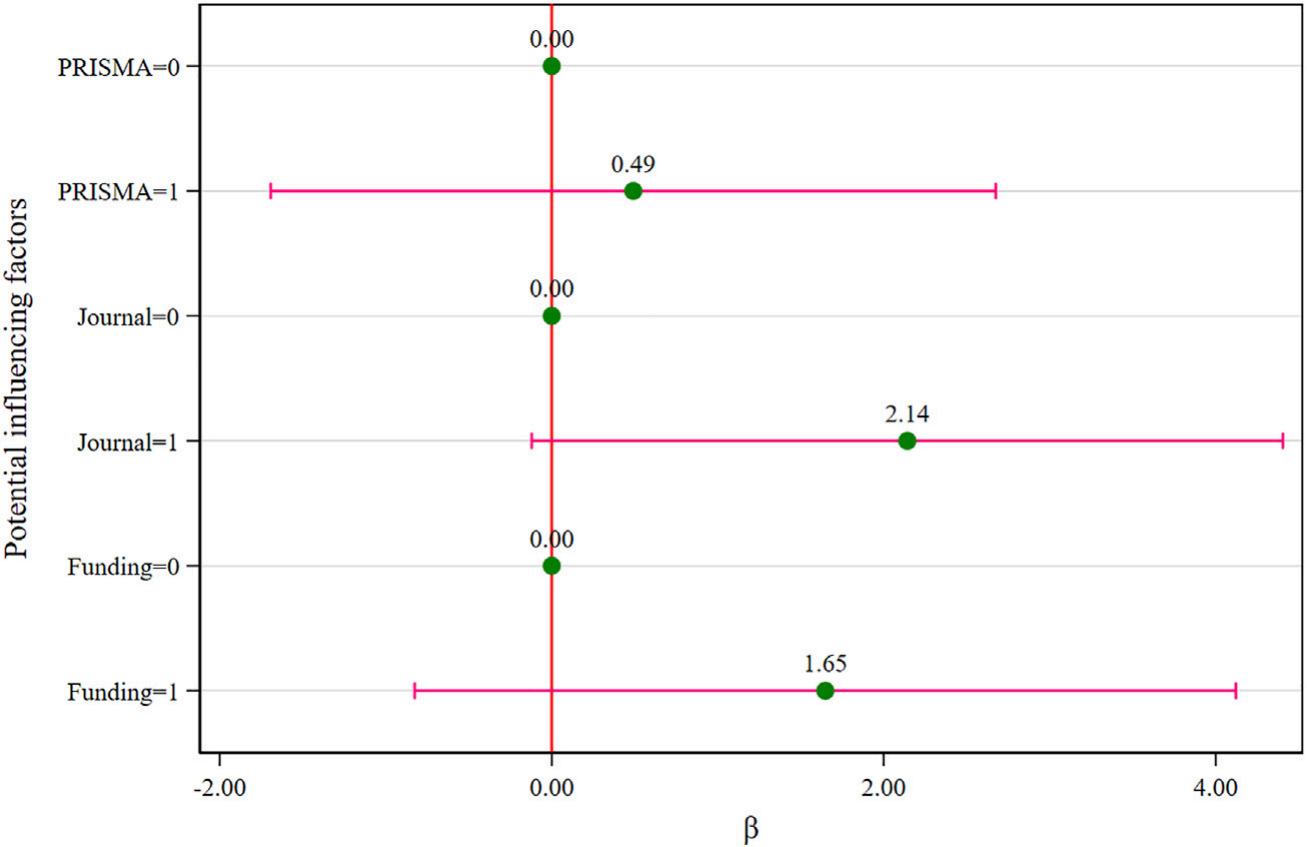
Multivariate analysis of the methodological quality score (the assignments see Table 2).

In addition, when compared to the reference group (Table 2), the exploratory results indicated that the journals’ IF (*β* = 2.81; 95%CI: 0.69 to 4.92; *p* = 0.012) and funds support (*β* = 2.68; 95%CI: 0.40 to 4.96; *p* = 0.024) were statistically significant in terms of the impact on the methodological quality score in the univariate analysis, but were not statistically significant (*p* = 0.062, *p* = 0.177) in the multivariate analysis (Figure 4).

**TABLE 2:** Univariate and multivariate analyses of the methodological quality score.

Study characteristics		Univariate analysis		Multivariate analysis	
		β (95% CI)	p	β (95% CI)	p
PRISMA	Not mentioned (=0)				
	Mentioned (=1)	1.20 (−1.25, 3.65)	0.317	0.49 (−1.69, 2.68)	0.640
Journal	IF ≤ 2 (=0)				
	IF > 2 (=1)	2.81 (0.69, 4.92)	0.012	2.14 (−0.12, 4.41)	0.062
Funding	No/not mentioned (=0)				
	With funds (=1)	2.68 (0.40, 4.96)	0.024	1.65 (−0.83, 4.12)	0.177

Note: IF, impact factor. The numbers in parentheses represent the assignments used in the model (VIF_max_ = 1.30).

### Pooled Health Outcomes on Efficacy

All included systematic reviews reported the pooled health outcomes on efficacy (Supplementary Material S4). These outcomes mainly included the tumor response rate (e.g., objective response rate, disease control rate), quality of life (e.g., Karnofsky performance status), survival rate (e.g., 1-year, 2-years survival rate), immune function (e.g., CD3^+^, CD4^+^/CD8^+^ ratio), and tumor pain relief, among others.

In terms of tumor response rate, the objective response rate was most commonly reported by the 17 MAs. Among them, 13 pairwise MAs reported statistically significant differences between CMs (e.g., AiDi, BJOE, FFKS, HuaChanSu, and SQFZ injections) combined with chemotherapy and chemotherapy alone. However, Wu *et al.* (Wu et al., 2019) demonstrated in 2019 that there was a difference only when XiaoAiPing injection was combined with XELOX compared to XELOX alone (4 RCTs, 409 patients; RR: 1.36, 95%CI: 1.10 to 1.70, I^2^ = 0%, random model). According to Yang *et al.* (Yang et al., 2013), similar results were observed when cinobufotalin was combined with chemotherapy compared to chemotherapy alone (7 RCTs, 448 patients; OR: 1.48, 95%CI: 1.01 to 2.07, I^2^ = 0%, fixed model); Li *et al.* (Li et al., 2020) reported no difference (4 RCTs, 254 patients; RR: 1.21, 95%CI: 0.99 to 1.21, I^2^ = 6%, fixed model) in the subgroup with a duration ≤4 weeks. Among the four network MAs, Wang *et al.* (Wang et al., 2014) reported that in a comparison between ten CM injections combined with FOLFOX and FOLFOX alone, the combinations of KangLaiTe, astragalus polysaccharides, cinobufacini, and BJOE with FOLFOX could improve the objective response rate the most. According to Zhang *et al.* (Zhang et al., 2017), a comparison between ten CM injections combined with FOLFOX and FOLFOX alone revealed that the combinations of astragalus, FFKS, KangAi, lentinan, HuaChanSu, and SQFZ with FOLFOX improved the objective response rate. Lu *et al.* (Lu X. et al., 2020) demonstrated that when seven oral Chinese patent medicines (e.g., BaZhen granule, Cinobufacin capsule, and XiaoAiPing tablet) combined with chemotherapy were compared to chemotherapy alone, the BaZhen granule combined with chemotherapy ranked first in terms of objective response rate. However, a network MA conducted by Zhang *et al.* (Zhang D. et al., 2018) showed no obvious difference between 11 CM injections (e.g., AiDi, KangAi, HuaChanSu, and FFKS) combined with XELOX compared to XELOX alone in terms of objective response rate.

Regarding the quality of life, the Karnofsky performance status was most frequently reported by the 16 MAs, and 12 pairwise MAs discovered statistical differences in this index between CMs (e.g., AiDi, BJOE, FFKS, HuaChanSu, and SQFZ injections) combined with chemotherapy compared to chemotherapy alone. For instance, when a recent MA by Cheng *et al.* (Cheng et al., 2021) found differences in the quality-of-life improvement regarding both the number of patients (14 RCTs, 939 patients; RR: 2.03, 95%CI: 1.70 to 2.43, I^2^ = 0%, random model) and the Karnofsky performance status score (6 RCTs, 329 patients; MD: 12.39, 95%CI: 5.48 to 19.30, I^2^ = 95%, random model) between CMs containing astragalus in combination with platinum-based chemotherapy and platinum-based chemotherapy alone.

Among the four network MAs, Wang *et al.* (Wang et al., 2014) reported that when nine CM injections combined with FOLFOX were compared to FOLFOX alone, the combinations of KangLaiTe, astragalus polysaccharides, cinobufacini, BJOE with FOLFOX could improve the performance status the most. A network MA published by Zhang *et al.* (Zhang et al., 2017) demonstrated that the combination of 11 CM (AiDi, SQFZ, FFKS, HuaChanSu, astragalus polysaccharides, KangAi, ginseng polysaccharide, lentinan, XiaoAiPing, and ShenMai) injections with FOLFOX could promote performance status compared to FOLFOX alone. In 2018, Zhang *et al.* (Zhang D. et al., 2018) performed another network MA and found that among the nine CM injections combined with XELOX, the SQFZ, HuaChanSu, KangAi, and BJOE injections could enhance the performance status compared to XELOX alone. A network MA published by Lu *et al.* (Lu X. et al., 2020) showed that the XiaoAiPing tablet in combination with chemotherapy significantly built up the performance status and ranked best among six oral Chinese patent medicines combined with chemotherapy when compared to chemotherapy alone.

As for the survival rate, four pairwise MAs reported a 1-year survival rate. However, only one (Cheng et al., 2021) among them demonstrated a statistical difference (8 RCTs, 512 patients; RR: 1.41, 95%CI: 1.09 to 1.82, I^2^ = 65%, random model) between CM containing astragalus combined with platinum-based chemotherapy compared to single platinum-based chemotherapy. Concerning immune function, both the CD3^+^ and the CD4^+^/CD8^+^ ratio were predominantly reported in four pairwise MAs, and all comparisons were statistically significant. Two MAs (Zhang X. et al., 2018; Sun et al., 2019) reported that the cinobufacini injection combined with chemotherapy statistically significantly alleviated tumor pain compared to chemotherapy alone. One MA (Chen et al., 2020) found a statistical difference between the SiJunZi decoction combined with enteral nutrition and enteral nutrition alone in terms of time to flatus (4 RCTs, 260 patients; MD: −9.45 h, 5%CI: −10.76 to −8.13, I^2^ = 0%, fixed model), length of hospital stay (3 RCTs, 200 patients; MD: −5.22 days, 95%CI: −7.46 to −2.99, I^2^ = 69%, random model), nutritional status (e.g., albumin, transferrin), and immune function (e.g., immunoglobulin A, immunoglobulin G) of postoperative gastric cancer patients. The detailed information on other efficacy outcomes is displayed in Supplementary Material S4.

### Pooled Clinical Outcomes on Safety

The pooled clinical outcomes on safety were reported in 18 MAs (Supplementary Material S4), which mainly included leucopenia, thrombocytopenia, adverse effects in the digestive system (e.g., nausea and vomiting, diarrhea), neurotoxicity, liver function damage (e.g., abnormal liver function, hepatic dysfunction, and hepatotoxicity), hand-foot syndrome, among others.

Leucopenia was the most commonly reported by 15 MAs, with 11 pairwise MAs demonstrating a significant effect between CMs (e.g., AiDi, BJOE, FFKS, HuaChanSu, and SQFZ injections) combined with chemotherapy and chemotherapy alone. However, Li *et al.* (Li et al., 2015) reported that the difference (6 RCTs, 363 patients; OR: 0.42, 95%CI: 0.23–0.70) was only found in the SQFZ injection combined with chemotherapy compared to chemotherapy alone for leucopenia (III-IV); Wu *et al.* (Wu et al., 2019) demonstrated that a statistically significant difference was identified only when the XiaoAiPing injection was combined with XELOX compared to XELOX alone (3 RCTs, 226 patients; RR: 0.68, 95%CI: 0.55 to 0.84, I^2^ = 0%, random model).

For the remaining four network MAs, Wang *et al.* (Wang et al., 2014) demonstrated that among the six CM injections combined FOLFOX, KangLaiTe, astragalus polysaccharides, cinobufacini, and BJOE combined with FOLFOX could reduce leucopenia (III-IV) compared to FOLFOX alone. The cluster analysis performed by Zhang *et al.* (Zhang et al., 2017) showed that the combination of astragalus polysaccharides with FOLFOX was the most effective in reducing leucopenia and gastrointestinal reactions. Furthermore, in 2018, their team (Zhang D. et al., 2018) demonstrated that among 13 CM injections combined with XELOX, lentinan, XiaoAiPing, and FFKS could significantly decrease leukopenia compared to XELOX alone. The network MA conducted by Lu *et al.* (Lu X. et al., 2020) showed that ZQFZ granule reduced leukopenia and ranked first among six CMs combined with chemotherapy compared to chemotherapy alone.

Thrombocytopenia was reported in seven pairwise MAs, with five reporting a statistical significance between CMs (e.g., AiDi, BJOE injections) combined with chemotherapy and chemotherapy alone. However, a fixed-effects MA (Sun et al., 2019) performed by Sun *et al.* reported no difference (356 patients; OR: 0.69, 95%CI: 0.44 to 1.11, I^2^ = 0%) in cinobufotalin combined with chemotherapy compared to chemotherapy alone. The same results were observed in the MA (Li et al., 2015) conducted by Li *et al.*, with no difference in SQFZ combined with chemotherapy compared to chemotherapy alone for thrombocytopenia (I-II) (4 RCTs, 259 patients; OR: 0.56, 95%CI: 0.29–1.08) or thrombocytopenia (III-IV) (3 RCTs, 197 patients; OR: 0.30, 95%CI: 0.06–1.50). In terms of adverse effects in the digestive system, the primary outcomes were nausea and vomiting, as well as diarrhea as reported by ten and 6 MAs, respectively. Among the 10 MAs reporting nausea and vomiting, seven pairwise MAs displayed a statistical difference between CMs combined with chemotherapy and chemotherapy alone. On the other hand, the other one pairwise MA (Wu et al., 2020) found no statistical difference (5 RCTs, 294 patients; RR: 0.81, 95%CI: 0.63 to 1.05, I^2^ = 0%, fixed model) on nausea and vomiting between the HuaChanSu injection combined with chemotherapy and chemotherapy alone.

According to a network MA (Wang et al., 2014) performed by Wang *et al.*, among the eight CM injections combined with FOLFOX, the KangLaiTe, astragalus polysaccharides, cinobufacini, and BJOE injections could reduce nausea and vomiting compared to FOLFOX. The other one network MA (Zhang D. et al., 2018) revealed that when comparing 13 CM injections combined with XELOX to XELOX alone, lentinan, disodium cantharidinate, and vitamin B6, SQFZ, and KangAi could significantly decrease nausea and vomiting. Among the six pairwise MAs that pooled data on diarrhea, four identified a statistical difference between CMs combined with chemotherapy and chemotherapy alone. However, Zhang *et al.* (Zhang X. et al., 2018) reported no significant effect (RR: 0.77, 95%CI: 0.52 to 1.15, I^2^ = 0%, fixed model) in the cinobufacini injection combined with chemotherapy compared to chemotherapy alone. Similarly, Wu *et al.* (Wu et al., 2020) similarly observed no difference (5 RCTs, 294 patients; RR: 0.86, 95%CI: 0.55 to 1.36, I^2^ = 0%, fixed model) in the HuaChanSu injection combined with chemotherapy compared to chemotherapy alone.

There were eight pairwise MAs that described neurotoxicity, with five demonstrating no statistical difference when CMs (e.g., BJOE, HuaChanSu, and SQFZ injections) combined with chemotherapy were compared to chemotherapy alone. Nevertheless, Chen *et al.* (Chen et al., 2018) reported a difference (5 RCTs, 356 patients; OR: 0.33, 95%CI: 0.20 to 0.55, I^2^ = 0%, fixed model) in JPBS therapy combined with chemotherapy compared to chemotherapy; the difference (528 patients; OR: 0.32, 95%CI: 0.20 to 0.50, I^2^ = 0%, fixed model) was also demonstrated by Sun *et al.* (Sun et al., 2019) when cinobufotalin combined with chemotherapy was compared to chemotherapy alone on peripheral neurotoxicity; similar results were observed by Cheng *et al.* (Cheng et al., 2021), where they validated a statistical difference (12 RCTs, 768 patients; RR: 0.78, 95%CI: 0.65 to 0.92, I^2^ = 0%, random model) between CMs containing astragalus combined with platinum-based chemotherapy and platinum-based chemotherapy on neurotoxicity. A pairwise MA (Chen et al., 2020) reported a statistical difference between the SiJunZi decoction combined with enteral nutrition compared to enteral nutrition alone regarding postoperative complications (2 RCTs, 110 patients; RR: 0.14, 95%CI: 0.03 to 0.64, I^2^ = 0%, fixed model).

Liver function damage was reported by eight systematic reviews, with four pairwise MAs identifying a statistical difference in CMs (e.g., AiDi, BJOE, and XiaoAiPing injections) combined with chemotherapy compared to chemotherapy alone. However, Wu *et al.* (Wu et al., 2019) only observed a difference when the XiaoAiPing injection was combined with XELOX compared to XELOX (3 RCTs, 226 patients; RR: 0.59, 95%CI: 0.37 to 0.92, I^2^ = 0%, random model). A network MA (Zhang et al., 2017) conducted by Zhang *et al.* revealed that the combination of disodium cantharidinate and vitamin B6 with FOLFOX was the most effective in reducing hepatic dysfunction and gastrointestinal reactions using cluster analysis. The remaining three (Li et al., 2015; Sun et al., 2019; Li et al., 2020) pairwise MAs demonstrated that no statistical differences in SQFZ combined with chemotherapy compared to chemotherapy alone, in CMs combined with paclitaxel-based chemotherapy compared to paclitaxel-based chemotherapy alone, and in cinobufotalin combined with chemotherapy compared to chemotherapy alone. Hand-foot syndrome was reported by six pairwise MAs, with four systematic reviews reporting a statistical difference between CMs combined with chemotherapy and chemotherapy alone. The other 2 MAs (Li et al., 2015; Wu et al., 2018) reported found no significant differences in SQFZ combined with chemotherapy compared to chemotherapy, and in BJOE combined with chemotherapy compared to chemotherapy alone (4 RCTs; RR: 0.78, 95%CI: 0.57 to 1.08, fixed model). The detailed information on other safety outcomes is displayed in Supplementary Material S4.

## Discussion

In this methodological investigation, we assessed and summarized current evidence from 16 pairwise and four network MAs focusing on CM as an adjunctive treatment for gastric cancer. Although the included MAs reported that the combination of CMs with other interventions (e.g., chemotherapy, enteral nutrition) could improve several clinical outcomes, the methodological quality of relevant MAs requires significant improvement.

Surgery and chemotherapy are regarded as the most important treatments for patients with gastric cancer. However, the toxicity of chemotherapy can lower function status and result in adverse drug reactions (Chen et al., 2020; Wang et al., 2020). In this study, a large number of CMs (e.g., AiDi, FFKS, HuaChanSu, KangAi, and SQFZ) combined with chemotherapy or the SiJunZi decoction combined with enteral nutrition were identified to potentially improve the efficacy of outcomes and reduce adverse effects. According to the theory of traditional CM (So et al., 2019), the occurrence of cancer is due to body function imbalance, which is usually caused by exopathogens, external environmental factors, improper diet, and emotional disorders; therefore, the main therapeutic principle of anti-cancer treatment is to restore balance by removing harmful factors, strengthening immunity, adjusting the flow of “Qi” and “Blood,” and softening hard tumors (So et al., 2019). Of note, the above-mentioned mechanisms and therapeutic methods are all applicable to gastric cancer. For example, the AiDi injection is prepared from the extracts of four CMs, including RenShen (Panax ginseng C.A.Mey. [Araliaceae]), HuangQi (Astragalus mongholicus Bunge [Fabaceae]), CiWuJia (Eleutherococcus senticosus (Rupr. and Maxim.) Maxim. [Araliaceae]), and BanMao (Mylabris phalerata Pallas) (Wang et al., 2015). According to the theory of traditional CM, this injection primarily clears heat, detoxifies the body, and eliminates blood stasis (Wang et al., 2015). In modern western medicine, it can induce apoptosis, inhibit tumor growth, and improve immune function (Wang et al., 2015). In China, FFKS has a long history of being used to treat gastric cancer and other tumors (Li et al., 2014; Yu et al., 2021). It is prepared from the extracts of KuShen (Sophora flavescens Aiton [Fabaceae]) and BaiTuLing (Heterosmilax japonica Kunth) and can clear heat and dampness, cool blood and detoxification, soften nodes, and relieve pain (Yu et al., 2021).

Previous findings have shown the anti-cancer properties of HuaChanSu through inducing cell differentiation and apoptosis, inhibiting cell proliferation, and reversing multi-drug resistance, among others (Xie et al., 2013). Although current evidence demonstrated that various CMs combined with chemotherapy were more effective than chemotherapy alone, most MAs stated that these findings required validation through well-conducted, large, multinational, multicenter RCTs with long-term follow-up. The main reasons were as follows: 1) the reporting and methodological qualities (e.g., methods of randomization, allocation concealment, and blinding) of included RCTs in MAs were not satisfactory according to the assessment results reported in the included MAs (Chen et al., 2020; Wu et al., 2020; Cheng et al., 2021), and the results of biased RCTs were not reliable; 2) survival time (especially the long-term survival rate) was a critical endpoint for cancer, but it did not receive significant attention (Zhang D. et al., 2018); 3) the evaluation and reporting of outcomes were inconsistent or messed up (Zhang X. et al., 2018), which hampered peer communication and restricted evidence synthesis (Alkhaffaf et al., 2021). Therefore, it is necessary to develop a core outcome set for RCTs on CMs for gastric cancer; 4) according to the included MAs (Zhang et al., 2017; Wu et al., 2019; Lu X. et al., 2020), almost all RCTs were conducted in China, so the clinical effects of CMs for patients with gastric cancer in other countries remain largely unexplored.

According to the AMSTAR-2 tool, the contents that required significant improvement were items 2 (study protocol and registration), 3 (explanation for the inclusion of study design), 7 (list of excluded studies with justifications), 8 (adequate details of included studies), 10 (funding sources of primary studies), and 12 (evaluation of the potential impact of risk of bias in primary studies on the synthesized results). The prospective registration of MAs’ protocol is an important process that can improve the transparency and reproducibility of the results (Page et al., 2018), while a recent meta-epidemiological study (Zheng et al., 2021) demonstrated that registered reviews of type 2 diabetes mellitus had a higher overall score of methodological quality. According to a previous study (Page et al., 2018), there were 26,535 records on the most used registration website for systematic reviews, PROSPERO, up until 10th October 2017. However, many researchers did not register the protocols or update registration records of their MAs, as revealed in this study and other publications (Tsujimoto et al., 2017; Rombey et al., 2020). Reviewers ignoring protocol registration was most possibly due to a lack of relevant knowledge and awareness on protocol and registration (Tawfik et al., 2020). Although an RCT is the gold standard for evaluating the clinical effects of interventions, harmful outcomes are constantly missed or the statistical power of trials is inadequate (Shea et al., 2017). However, non-randomized interventional studies could address these issues (Shea et al., 2017). A systematic review with MA should present an entire landscape of outcomes of interest, hence the AMSTAR-2 requires the reviewers to justify their study design selections in their reviews (Shea et al., 2017). In addition, a methodological overview (Golder et al., 2011) suggested that systematic reviews of adverse reactions should not restrict the inclusion of specific study designs.

Item 7 of AMSTAR-2 expects the authors to provide a complete list of excluded publications and the reasons for their exclusion at the full-text screening stage, which can improve the transparency of the selection process and aid in judging the completeness of the results. Some studies (DeAngelis and Fontanarosa, 2008; Lundh et al., 2018) showed that having no independent funds support may be linked to financial conflicts of interest; for example, the authors may present favorable outcomes and/or overstate the effects of drugs or devices provided by industry funders. Low-quality RCTs with a high risk of bias may distort the pooled outcomes reported in MAs; therefore, authors should investigate the potential impact of the risk of bias in RCTs on the results of MAs (Shea et al., 2017). When assessing the methodological quality of included primary studies, the AMSTAR-2 suggested using the updated Cochrane tool for RCTs and the ROBINS-I (Schwingshackl et al., 2021) tool for non-randomized interventional studies. As previously mentioned, the reporting and methodological quality of relevant RCTs require improvement, and we recommend authors conducting RCTs to follow the updated Cochrane tool (Schwingshackl et al., 2021), CONSORT Chinese Herbal Medicine Formulas 2017 guidelines (Cheng et al., 2017), and CONSORT 2010 (Schulz et al., 2010) for designing and reporting Chinese herbal medicine formula trials. Doing so because not only trials should be well-designed and conducted but also assessment of bias in primary studies in MAs is usually only completed based on methodology aspects reported by the trialists (Whiting et al., 2017). Therefore, it is hard to assess the “true methodology,” if a study is not reported with adequately relevant details (Pollock et al., 2017; Whiting et al., 2017). Similarly, this is not inception for MA; reviewers should not only implement an MA according to a high methodology standard but also report all relevant methodology details clearly and completely; thus, the authors of overviews of MAs can acquire the adequate information to assess the quality of MAs (Glasziou et al., 2008; Pollock et al., 2017).

Publication bias mainly includes the selective publication of studies or selective reporting of results, which can affect the estimates of interventions by overstating efficacy or diluting safety (Furuya-Kanamori et al., 2020). In this overview, the percentage of “Y” in item 15 was just equal to our predefined threshold. Therefore, the investigation and discussion of potential publication bias of the MAs included in this overview required improvements. To this end, there are many statistical methods (e.g., Egger’ test, Begg’ test, and LFK index) (Furuya-Kanamori et al., 2020) available for judging publication bias except for the funnel plot. In addition, although positive effects of funds support and journals’ IF on the methodological quality score were identified in the univariate analysis, they were not statistically significant in the multivariate analysis, potentially due to the limited number of MAs. However, a cross-sectional study (Xu et al., 2019) published in 2019, included 529 dose-response MAs and found that publications receiving financial support had higher methodological quality scores (based on the modified AMSTAR tool) than those without funds or that did not report funding information. Furthermore, the methodological research conducted by Fleming *et al.* (Fleming et al., 2014), reported that among 372 interventional systematic reviews, those published in clinical journals with higher IF appeared to have better methodological quality based on the AMSTAR tool. In general, rigorous peer-review and publication processes can improve the quality of publications (Rice et al., 2021), and high-IF journals frequently adhere to the above processes. The research with funds support often needs to be strictly reviewed and assessed by their funders, which may potentially affect the methodological quality of MAs included in this systematic investigation.

To the best of our knowledge, this is the first study using the evidence mapping method to visualize the methodological quality on published MAs of CM as an adjunctive treatment for gastric cancer. In addition, evidence on the efficacy and safety of CM for patients with gastric cancer was summarized and compared to the pooled results across multiple MAs. Furthermore, the potential factors affecting the methodological quality score of the included MAs were investigated, and the results were displayed using a forest plot. However, there were several limitations to this study. First, two commonly used databases were searched and only MAs published in English were included rather than other languages, such as Chinese, which may limit the generalizability of the results. However, a recent study (Cao et al., 2021) demonstrated that the methodological quality of MAs published by Chinese researchers in English was slightly higher than those published in Chinese. Second, the results of regression analyses may be biased due to the limited number of included MAs; however, results from other similar publications (Fleming et al., 2014; Xu et al., 2019) provided evidence support for our study.

## Conclusion

As the first evidence mapping study on MAs of CMs for gastric cancer, we identified that published MAs demonstrated various CMs (e.g., AiDi, FFKS, and HuaChanSu) in combination with chemotherapy which can potentially improve efficacy (e.g., objective response rate, quality of life, immune function) and reduce adverse reactions (e.g., leucopenia, thrombocytopenia, nausea and vomiting). However, the methodology (e.g., study protocol and registration, explanation for study design inclusion, reporting on funding sources of RCTs) of relevant MAs requires significant improvement, and more methodologically robust RCTs are needed.

## Data Availability

The original contributions presented in the study are included in the article/Supplementary Material, further inquiries can be directed to the corresponding authors.
