# Supplementary material for: Chinese Medicine as an Adjunctive Treatment for Gastric Cancer: Methodological Investigation of meta-Analyses and Evidence Map
Source: Front Pharmacol. 2022 Jan 10;12:797753. doi: 10.3389/fphar.2021.797753 (PMC8784830; doi:10.3389/fphar.2021.797753)
Supplement: Supplementary file 3 [file Table2.docx]

**Search strategy (1^st^ September, 2021)**

**PubMed (records=57)**

***#1 /OR***

"Medicine, Chinese Traditional"[Mesh]

"Complementary Therapies"[Mesh]

"Chinese materia medica"[Title/Abstract]

"Chinese medicine"[Title/Abstract]

"Chinese medicines"[Title/Abstract]

"Traditional medicine"[Title/Abstract]

"Traditional medicines"[Title/Abstract]

herb*[Title/Abstract]

pill*[Title]

formula*[Title]

granule*[Title]

injection*[Title]

decoction[Title]

Wan[Title]

San[Title]

Gao[Title]

Pian[Title]

Dan[Title]

Yin[Title]

Ji[Title]

***#2 /OR***

"Stomach Neoplasms"[Mesh]

"gastric cancer"[Title/Abstract]

"gastric cancers"[Title/Abstract]

"gastric carcinoma"[Title/Abstract]

"gastric carcinomas"[Title/Abstract]

"gastric adenocarcinoma"[Title/Abstract]

"gastric adenocarcinomas"[Title/Abstract]

"gastric neoplasm"[Title/Abstract]

"gastric neoplasms"[Title/Abstract]

"gastric tumour"[Title/Abstract]

"gastric tumours"[Title/Abstract]

"stomach cancer"[Title/Abstract]

"stomach cancers"[Title/Abstract]

"stomach carcinoma"[Title/Abstract]

"stomach carcinomas"[Title/Abstract]

"stomach adenocarcinoma"[Title/Abstract]

"stomach adenocarcinomas"[Title/Abstract]

"stomach neoplasm"[Title/Abstract]

"stomach neoplasms"[Title/Abstract]

"stomach tumor"[Title/Abstract]

"stomach tumors"[Title/Abstract]

***#3 /OR***

"Systematic Review"[Publication Type]

"Systematic Reviews as topic"[Mesh]

"Meta-analysis"[Publication Type]

"Meta-analysis as topic"[Mesh]

"systematic review"[Title/Abstract]

"meta-analysis"[Title/Abstract]

***#4 #1 AND #2 AND #3***

**Embase (records=61)**

***#1 /OR***

'Chinese medicine'/exp

'Chinese materia medica':ab,ti

'Chinese medicine':ab,ti

'Chinese medicines':ab,ti

'Alternative medicine':ab,ti

'Alternative medicines':ab,ti

'Complementary medicine':ab,ti

'Complementary medicines':ab,ti

'Traditional medicine':ab,ti

'Traditional medicines':ab,ti

herb*:ti

pill*:ti

formula*:ti

granule*:ti

injection*:ti

decoction:ti

Wan:ti

San:ti

Gao:ti

Pian:ti

Dan:ti

Yin:ti

Ji:ti

***#2 /OR***

'stomach cancer'/exp

'gastric cancers':ab,ti

'gastric carcinoma':ab,ti

'gastric carcinomas':ab,ti

'gastric neoplasm':ab,ti

'gastric neoplasms':ab,ti

'gastric tumour':ab,ti

'gastric tumours':ab,ti

'stomach cancer':ab,ti

'stomach cancers':ab,ti

'stomach carcinoma':ab,ti

'stomach carcinomas':ab,ti

'stomach neoplasm':ab,ti

'stomach neoplasms':ab,ti

'stomach tumor':ab,ti

'stomach tumors':ab,ti

***#3 /OR***

'systematic review'/exp

'meta analysis'/exp

'systematic review':ab,ti

'meta-analysis':ab,ti

***#4 #1 AND #2 AND #3***
